# Source and dynamics of a volcanic caldera unrest: Campi Flegrei, 1983–84

**DOI:** 10.1038/s41598-017-08192-7

**Published:** 2017-08-14

**Authors:** Luca De Siena, Giovanni Chiodini, Giuseppe Vilardo, Edoardo Del Pezzo, Mario Castellano, Simona Colombelli, Nicola Tisato, Guido Ventura

**Affiliations:** 1 University of Aberdeen, School of Geosciences, Dept. Geology and Petroleum Geology, Meston Building, King’s College, Aberdeen, AB24 3UE Scotland UK; 2 Istituto Nazionale di Geofisica e Vulcanologia, Sezione di Bologna, Via D. Creti 12, 40128 Bologna, Italy; 3 Istituto Nazionale di Geofisica e Vulcanologia, Sezione di Napoli-Osservatorio Vesuviano, Via Diocleziano 328, 80124 Napoli, Italy; 4 Instituto Andaluz de Geofisica, Universidad de Granada, Calle Prof. Clavera, Campus Universitario de Cartuja, Granada, Spain; 5 Department of Physics, University of Naples Federico II, Napoli, Italy; 6 The University of Texas at Austin, Jackson School of Geosciences, Department of Geological Sciences, 2275 Speedway Stop C9000, Austin, TX 78712 USA; 7 Dept. of Civil Engineering, University of Toronto, 35 St. George St., M5S 1A4, Toronto, Ontario Canada; 8 Istituto Nazionale di Geofisica e Vulcanologia, Sezione di Roma, Via di Vigna Murata 605, 00181 Roma, Italy; 9 Istituto per l’ Ambiente Marino Costiero, CNR, Napoli, Italy

## Abstract

Despite their importance for eruption forecasting the causes of seismic rupture processes during caldera unrest are still poorly reconstructed from seismic images. Seismic source locations and waveform attenuation analyses of earthquakes in the Campi Flegrei area (Southern Italy) during the 1983–1984 unrest have revealed a 4–4.5 km deep NW-SE striking aseismic zone of high attenuation offshore Pozzuoli. The lateral features and the principal axis of the attenuation anomaly correspond to the main source of ground uplift during the unrest. Seismic swarms correlate in space and time with fluid injections from a deep hot source, inferred to represent geochemical and temperature variations at Solfatara. These swarms struck a high-attenuation 3–4 km deep reservoir of supercritical fluids under Pozzuoli and migrated towards a shallower aseismic deformation source under Solfatara. The reservoir became aseismic for two months just after the main seismic swarm (April 1, 1984) due to a SE-to-NW directed input from the high-attenuation domain, possibly a dyke emplacement. The unrest ended after fluids migrated from Pozzuoli to the location of the last caldera eruption (Mt. Nuovo, 1538 AD). The results show that the high attenuation domain controls the largest monitored seismic, deformation, and geochemical unrest at the caldera.

## Introduction

**Figure 1:**
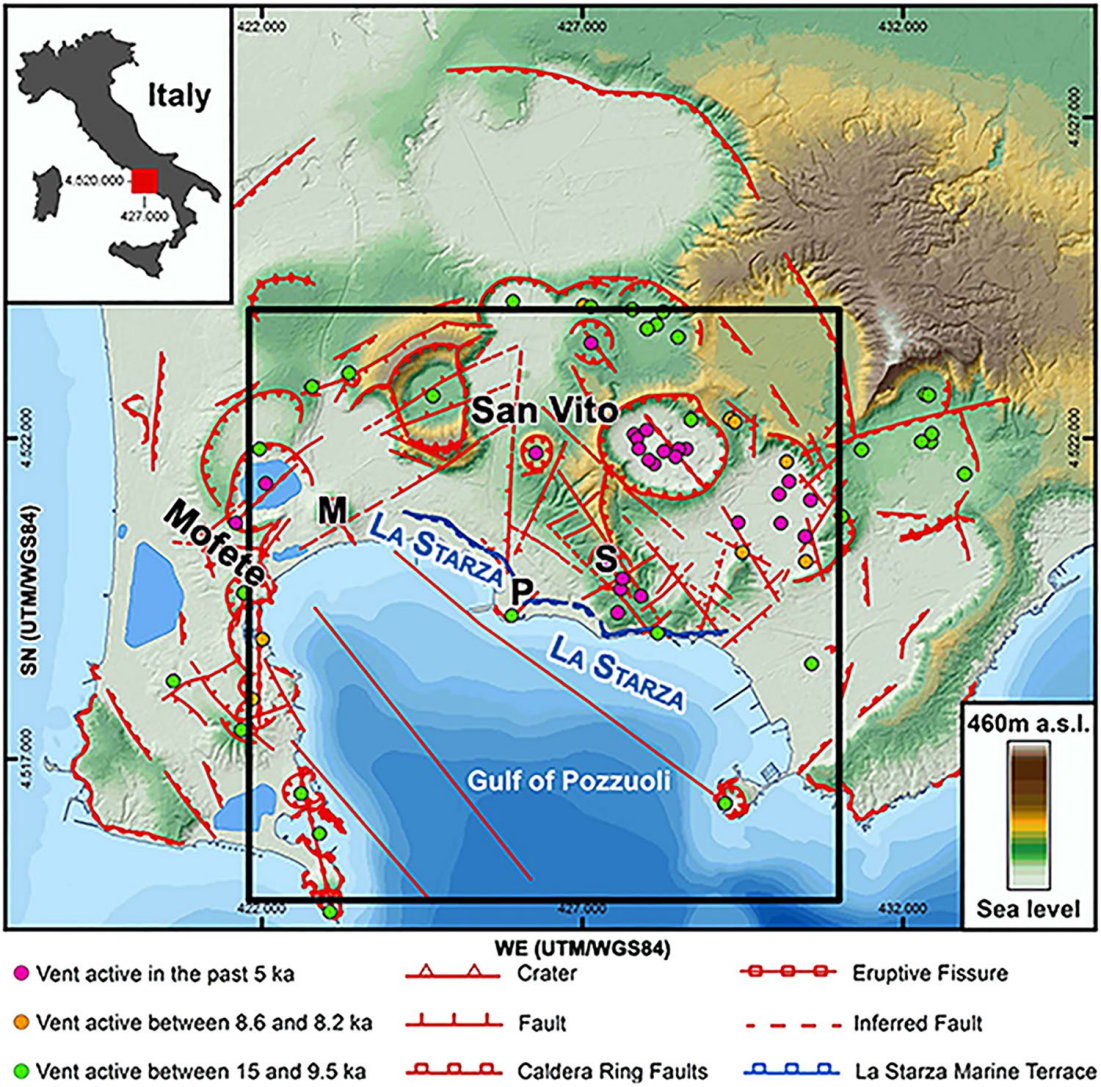
Structural map of Campi Flegrei caldera. The upper left panel shows the caldera location in Italy. Bold letters mark the volcanic centres of Pozzuoli, Solfatara, and Mt. Nuovo. The Naples metropolitan area extends east of Solfatara. Major faults are marked by red lines and volcanic vents coloured by age. Redrawn joining the results in Vilardo *et al*.^31^ and Vitale *et al*.^30^ using ESRI ArcGIS 10.0 (https://www.esri.com/training/catalog/5763042b851d31e02a43ed4d/using-arcmap-in-arcgis-desktop-10/). Faults and coastline are transformed into a shape file in this workspace and imported in Voxler 3.0^©^ (http://www.goldensoftware.com/products/voxler). They are then imposed on all maps reproduced in the following figures.

Campi Flegrei caldera (South Italy, Fig. 1) consists of two nested calderas related to two main eruptive events with volcanic explosive indexes greater than five (Campanian Ignimbrite - ~39 kA and 18–20 km diameter - and Neapolitan Yellow Tuff - ~15 kA and 7–10 km diameter). The caldera is situated in a Pliocene-Quaternary extensional domain of NE-SW and NW-SE trending normal faults of the Tyrrhenian margin of the Apennine thrust belt^1^. During the Holocene, the region has been subjected to a tectonic ESE-WNW extension^2^. The last eruption occurred in 1538 AD (Mt. Nuovo, Fig. 1 - labelled by **M**), NW of the caldera centre^3^. Historical, archaeological and geological records show that the eruption was preceded by regional uplift and earthquakes, magma accumulation in a 4.6 ± 0.9 km deep source below the caldera centre, and magma transfer to a 3.8 ± 0.6 km deep magmatic source ~4 km below Mt. Nuovo^4^. An eruption of similar scale would be highly destructive for the dense metropolitan city of Naples (about 3.1 million inhabitants), which comprises the caldera.

In the last 50 years, two volcanic unrests (1969–72 and 1982–84) have been monitored using seismic, deformation, and geochemical data. The 1982–84 deformation unrest produced a net ground uplift of 1.8 m^2, 5, 6^ measured near the city of Pozzuoli (labelled by **P** in Fig. 1). Deformation studies agree that the main deformation source intersected Pozzuoli^5, 7–10^, but they generally disagree on its nature (magmatic^11^ or produced by hydrothermal systems and decarbonation reactions^12–14^), depth, and extension. During the unrest, levelling and gravimetric data show that the main deformation source can be modelled as a penny-shaped, 2500 *kg*/*m* ^3^ magmatic source between depths of 3 and 4 km, located just offshore Pozzuoli^8^. The pattern of deformation was also consistent with the intrusion of magma of 3 m thickness as a sill in this depth range and connected to the simultaneous strain of the crust in the ESE-WNW direction^2^. A quasi-horizontal elongated crack oriented NW-SE and centred offshore Pozzuoli was the main source of uplift during the 1980–2010 ground displacements^11^. The crack is schematised as a laterally extended pressurised triaxial ellipsoid of 100 m thickness at a depth of 3.6 km. Between 1980 and 2010, it was paired with a stationary point source, acting below Solfatara at a depth of 1.9 km^11^.

**Figure 2:**
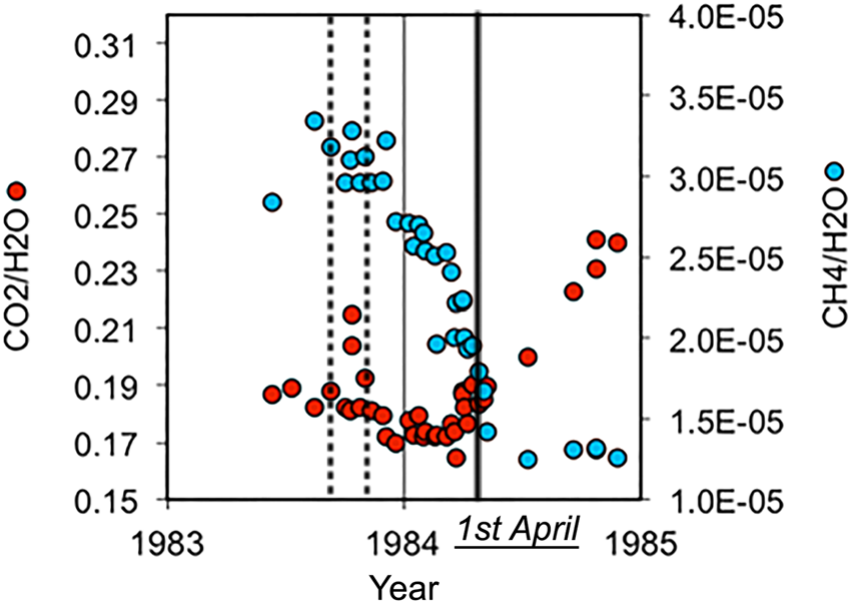
Geochemical gas composition during unrest. *CO*_2_/*H*_2_ *O* (left, red dots) and *CH*_4_/*H*_2_ *O* (right, blue dots) ratios measurements against time. April 1, when the decrease of *CH*_4_/*H*_2_ *O* stops and the increase of *CO*_2_/*H*_2_ *O* starts, is marked by a black bar. September-October 1983 (between the vertical dashed lines) corresponds to the period when fluid injections are perturbing the upper hydrothermal system at Solfatara, following the fluid-flow modelling of Chiodini *et al*.^17^. The plot is obtained using the plotting functions of Microsoft Excel^©^ for Mac 2011 14.4.9 (https://www.microsoft.com/en-us/download/details.aspx?id=46571).

The Solfatara (Fig. 1, **S** - East of Pozzuoli) and Mofete/Mt. Nuovo (**M** - West of Pozzuoli) fumaroles provided most of the data used to study geochemical unrests (strong variations in the temporal trends of geochemical data) at the caldera^12, 13, 15, 16^. Figure 2 shows the temporal variations in the concentration of *CO*_2_/*H*_2_ *O* and *CH*_4_/*H*_2_ *O* ratios collected at Solfatara between May 1983 and December 1984^15^. Until (or just before) April 1, 1984, a slow decrease in concentration of *CO*_2_/*H*_2_ *O* was paired with a steep decrease in *CH*_4_/*H*_2_ *O* concentration. After this date, the *CO*_2_/*H*_2_ *O* ratio drastically increased while the *CH*_4_/*H*_2_ *O* ratio remained constant until the end of 1984. These data were recently used to model a hot source of vertical fluid injections deeper than 2 km, acting between September and October 1983 (dashed lines in Fig. 2) and causing the main flux variations in the hydrothermal system during the unrest^17^. The isotopic study of the origin of sulphur, carbon, and methane in Solfatara fumaroles during the unrest showed that geochemical patterns for sulphur and carbon are inconsistent with magmatic sources shallower than 4 km acting in the same period^16, 18^.

After September 1983, the increase in number and intensity of shallow (<4.5 km depth) earthquakes in the centre of the caldera led the local authorities to evacuate >40000 people from the Pozzuoli City centre. Of these more than 20000 were permanently relocated to the new Monteruscello District, north of the city^19^. The average spatial seismic distribution and source properties during the 1982–84 unrest have been extensively studied and compared with geochemical and deformation data^12, 16, 20, 21^. Seismic intensities showed no clear correlations with ground uplift, while focal mechanisms were compatible with double couple source models^22, 23^.

**Figure 3:**
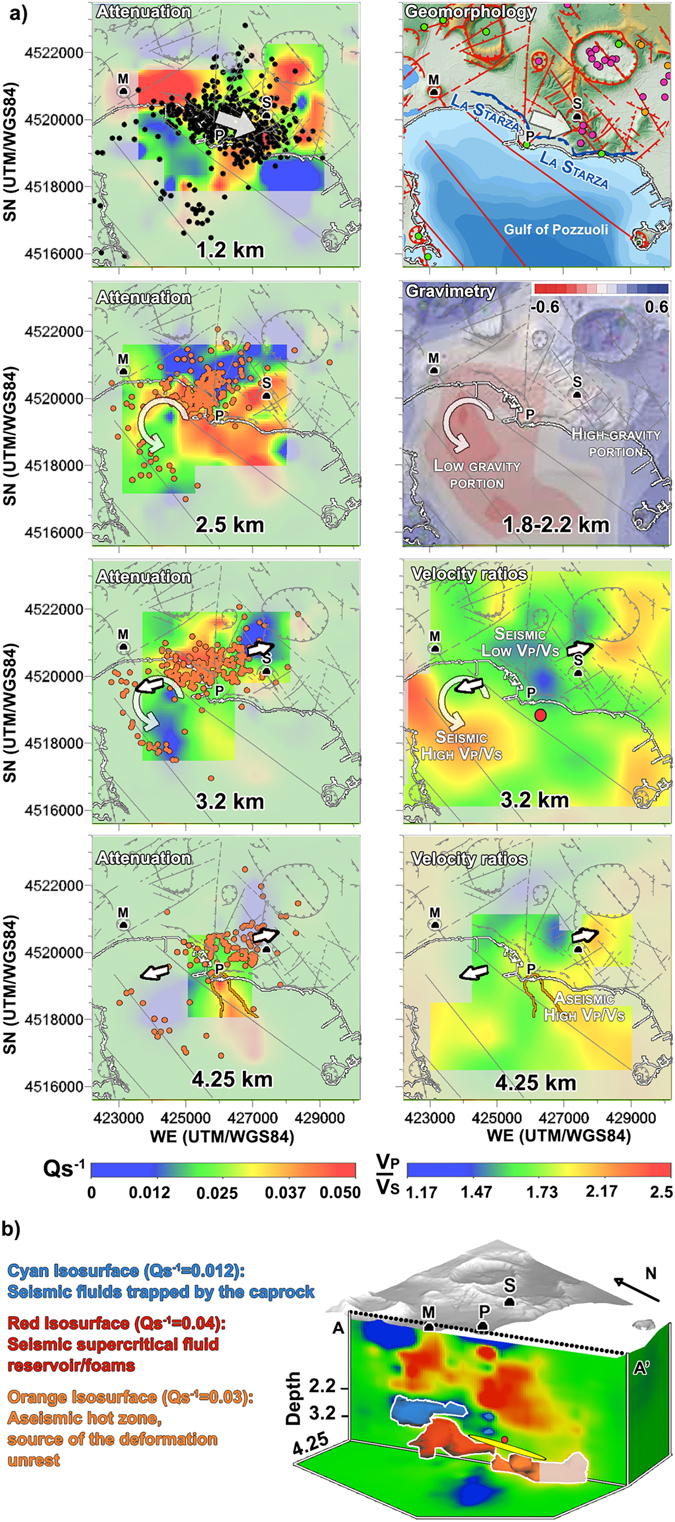
Comparison of the 3D attenuation and seismicity models with tectonics, gravimetry, and $\frac{{V}_{p}}{{V}_{s}}$ ratios. The uniformly coloured areas mark poorly resolved regions in the attenuation and $\frac{{V}_{p}}{{V}_{s}}$ models. (**a**) Left hand column: horizontal cross-sections into the 3D S-wave attenuation model at depths of 1.2, 2.5, 3.2 and 4.25 km. The seismicity (black circles) between 0.5 km and 2.2 km (depth of the caprock under Pozzuoli^14^) is plotted on the 1.2 km panel. Below the caprock, the seismicity 300 m above and below the depth of the tomogram is plotted as orange circles. The white arrows show earthquake propagation as deduced by the time dependent seismic patterns in Figs 4 and 5, and S6. Right column: the main earthquake propagation directions (white arrows) are superimposed on structural map and volcanic vents^30, 31^ (first row, obtained with ESRI ArcGIS 10.0^©^ (https://www.esri.com/training/catalog/5763042b851d31e02a43ed4d/using-arcmap-in-arcgis-desktop-10/), gravimetric anomalies recorded after the unrest (1.8–2.2 km^32^), and $\frac{{V}_{p}}{{V}_{s}}$ ratios (3.2 and 4.25 km^24^). The red circle in the 3.2 km $\frac{{V}_{p}}{{V}_{s}}$ panel delineates the penny-shaped, 2500 *kg*/*m* ^3^ magmatic anomaly obtained by Amoruso *et al*.^8^ from data recorded during the 1982–84 unrest. The resolved 4.25 km deep high-attenuation anomaly contour(${Q}_{S}^{-1}=0.03$) is imposed on the 4.25 km $\frac{{V}_{p}}{{V}_{s}}$ panel. (**b**) A 3D volumetric image and interpretation of the 3 main attenuation anomalies below the caprock. The unresolved part of the deepest anomaly is shaded with grey. The vertical cross-section AA’ cuts the 4–4.5 km deep high-attenuation zone and the main deformation anomalies (the yellow ellipse is the quasi-horizontal elongated crack that satisfies large-scale deformation^11^). Attenuation maps, $\frac{{V}_{p}}{{V}_{s}}$ maps, microseismic hypocentres, and isosurfaces were obtained using Voxler 3.0^*c*^ (http://www.goldensoftware.com/products/voxler) using the 3D data provided in the submission, the velocity model^24^, a distant weighting interpolation method of second order for tomograms. The layout of the figure, the axes for each panels, the deformation/gravity anomalies, and the arrows have been created or redrawn using Photoshop CS^©^ (https://helpx.adobe.com/uk/x-productkb/policy-pricing/cs6-product-downloads.html). Deformation/gravity anomalies reproduce the general features of the anomalies described by the corresponding studies^8, 11, 32^.

The seismic recordings produced by the SERAPIS active seismic experiment have been used to image crustal structures down to 8 km depth^9, 24, 25^ and have depicted few seismic structures spatially related to ground deformation or magmatic sources. Large amplitude seismic reflections show the top of a fluid-bearing rock formation extending across the caldera at depths of about 3–3.5 km^25^. These are interpreted as a “basement top”, as defined by the study of rock physical properties on Campi Flegrei samples^14^. A second smaller-amplitude reflection constrained a ~7.5 km deep, 1-km-thick, low-velocity layer, associated with a mid-crust, partially molten zone beneath the caldera^25^, compatible with the deepest magmatic source modelling the geochemical unrest^16, 18^. Between depths of 3 and 4 km, the lowest tomographically-derived $\frac{{V}_{p}}{{V}_{s}}$ ratios that marked the volumes below Pozzuoli (Fig. 3a, right-hand column, $\frac{{V}_{p}}{{V}_{s}}$) were embedded into the basement. They were sealed by a 1–2 km thick layer serving as a caprock^9, 14^, with a maximum depth of 2 km under Pozzuoli. In the interpretation of Vanorio and Kanitpanyacharoen (2015)^14^, at Campi Flegrei the characteristics of the basement and the high strength of the caprock allow *CO*_2_ to form and exert pressure and produce fluid-induced uplift under Pozzuoli. However, the structures producing the highest deformation and density anomalies during the 1982–84 unrest were located just offshore Pozzuoli^8, 11^. They strongly affected the seismic coda wave attenuation and the derived imaging. De Siena *et al*.^26^ delineated a circular, low-attenuation anomaly offshore Pozzuoli of 1 km diameter, similar to those imaged at other volcanoes^27^. This circular anomaly intersects the oldest vent active in the centre of the caldera (Fig. 1, green dot under Pozzuoli) and is interpreted as the remnant of previous eruptive activity. Finally, De Siena *et al*.^28^ used coda-normalised body-wave attenuation tomography and detected a high-attenuation 3–4 km deep anomaly under Pozzuoli. The high-attenuation domain was located in a zone of average $\frac{{V}_{p}}{{V}_{s}}$ ^9^ (about 1.73) and comprised both the area of maximum deformation and the main coda-wave attenuation anomaly^8, 26^.

**Figure 4:**
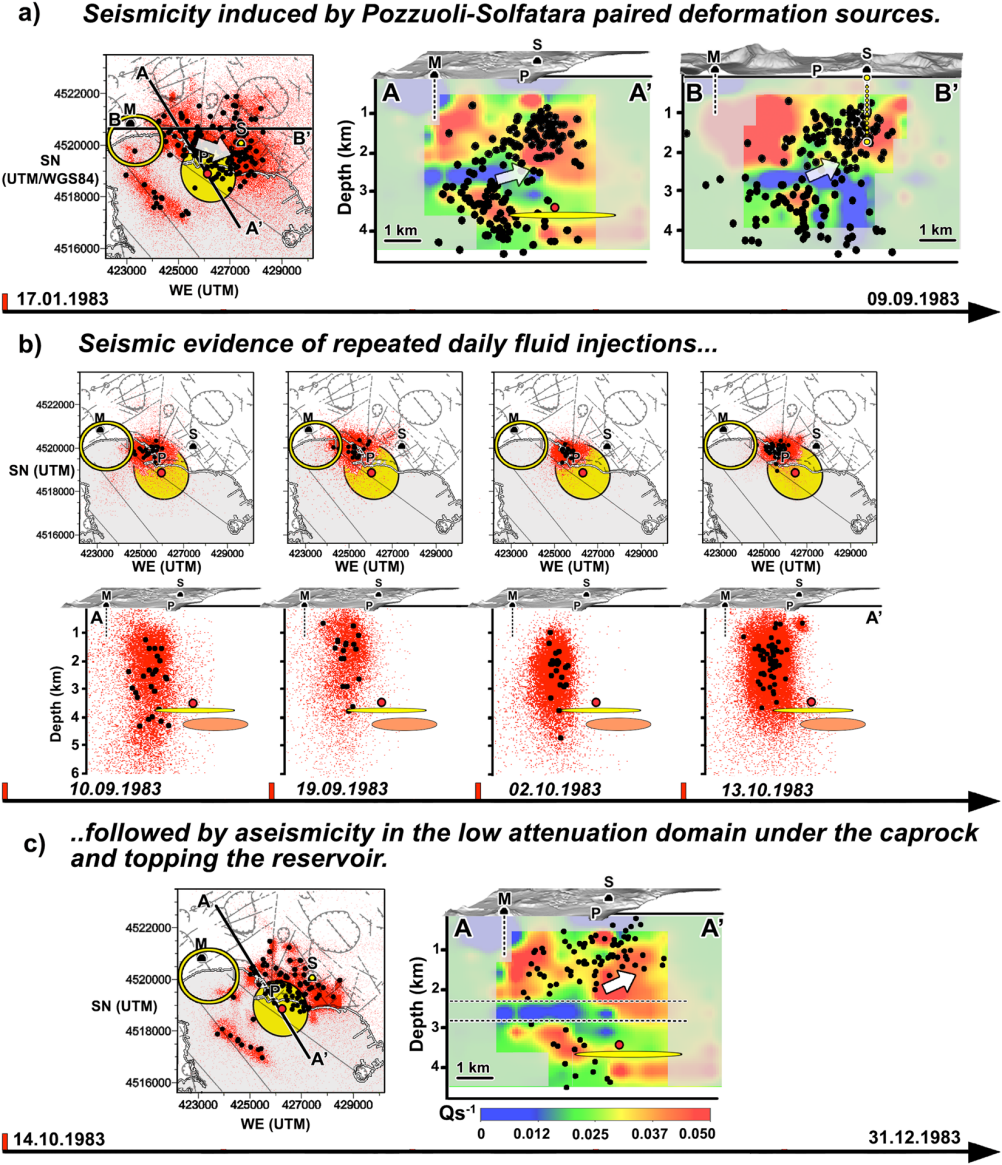
Time-dependent seismicity during the 1983 unrest. Microearthquakes (black circles) and corresponding probability density functions (PDF, red dots, 1000 samples per event) are drawn under the structural map of Campi Flegrei caldera (grey lines on each map) and on two vertical planes crossing the S-wave attenuation model (AA’ and BB’). All UTM coordinates use the WGS84 geodetic datum. The yellow circumference intersecting Mount Nuovo corresponds approximately to the end of the path followed by magma transfer and preceding the last eruption of the volcano^4^. All panels show the correlation of seismic and attenuation patterns with deformation sources. The sections of the main deformation ellipsoid (yellow ellipses) and magmatic anomaly (red circle) are shown on all maps and AA’ cross-sections. The small yellow circle on the maps and the BB’ cross-sections under Solfatara is the paired secondary deformation source^11^ active under Solfatara. Panel (a): The white arrow outlines the seismic propagation from Pozzuoli to Solfatara inside the caprock before September-October 1983. Panel (b) shows the seismic signature of the fluid injections modelled by Chiodini *et al*.^17^ using geochemical data. The 4–4.5 deep attenuation anomaly is approximated by an orange ellipse on all vertical sections. On a map, the resolved high-attenuation anomaly is concealed by the deformation anomaly. The figures were created using the same softwares and data described in Fig. 3 (ESRI ArcGIS 10.0^©^, https://www.esri.com/training/catalog/5763042b851d31e02a43ed4d/using-arcmap-in-arcgis-desktop-10/; Voxler 3.0^©^, http://www.goldensoftware.com/products/voxler; Photoshop CS^©^, https://helpx.adobe.com/uk/x-productkb/policy-pricing/cs6-product-downloads.html. The PDF are created using SeismicityViewer (http://alomax.free.fr/nlloc/) and imposing the results on each panel).

**Figure 5:**
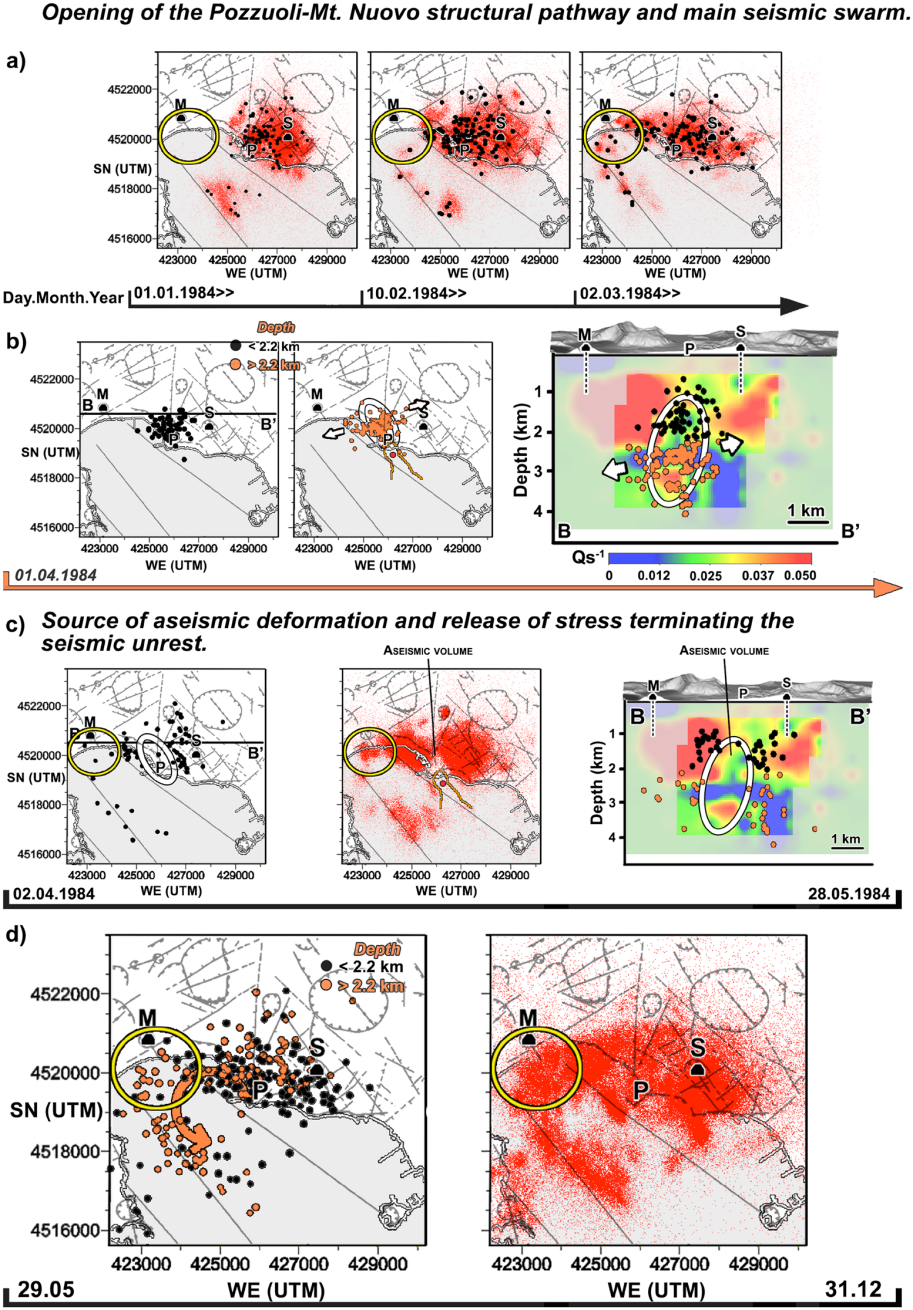
Time-dependent seismicity during the 1984 unrest. The symbols are the same used for Fig. 4. An isoline corresponding to ${Q}_{s}^{-1}=0.03$ is plotted on all maps between January and May and depicts the 4–4.5 deep high-attenuation anomaly. Panel (a) shows how the microseismicity progressively crosses the location of the last eruption, mainly following La Starza marine terrace. Panel b depicts the microearthquakes recorded on April 1: in this case, black (orange) circles are earthquakes recorded above (below) the maximum depth of the caprock^14^. The white ellipsoidal contour on the map and BB’ vertical section shows the area of densest microseismicity. In panel c, this same area becomes aseismic for 2 months. Almost no maximum likelihood hypocentres is located in the ellipsoid (left and right plots) while the PDF show a clear gap under Pozzuoli (central plot). Panel d depicts the end of the seismic unrest, with earthquakes repeatedly crossing the are of Mount Nuovo below the caprock (orange dots, see also the PDF) and following a NNW-SSE striking fault offshore^21, 31^. The figure wa created using the same softwares and data described in Fig. 4 (ESRI ArcGIS 10.0^©^, https://www.esri.com/training/catalog/5763042b851d31e02a43ed4d/using-arcmap-in-arcgis-desktop-10/; Voxler 3.0^©^, http://www.goldensoftware.com/products/voxler; Photoshop CS^©^, https://helpx.adobe.com/uk/x-productkb/policy-pricing/cs6-product-downloads.html; SeismicityViewer, http://alomax.free.fr/nlloc/.

Here, we present an updated seismic S-wave attenuation model, obtained by applying the MuRAT code^29^ to relocated microearthquakes recorded during the January-April 1984 seismic unrest (Figs 3, 4 and 5, attenuation tomograms). The new ray geometry increases in-depth illumination (down to 4.5 km, the previous limit was at 4 km^28^, see Figs S2–S5 for the corresponding resolution). The microearthquakes between January 1983 and December 1984 (Figs 3, 4 and 5, black and orange dots) are located in the 3D P- and S-wave velocity models of Battaglia *et al*.^24^ using the NonLinLoc software, catalog data pickings, pickings from the University of Wisconsin waveforms^20, 28^, and strict selection criteria (see methods). The probability density functions (PDF) corresponding to the hypocentres better (1) specify location uncertainties (see Methods section), providing a direct image of the volumes where the location is feasible and (2) quantify temporal changes in seismicity patterns, especially the occurrence of seismic swarms and the shape of “aseismic” regions (red dots in Figs 4 and 5, in particular Fig. 5b,c), where “aseismicity” is also defined with respect to the above-mentioned selection criteria. Additionally, we improve the estimates of the coda-normalised S-wave energies produced by the main seismic swarms crossing Pozzuoli by correcting for their source directivity (SE-NW, Figs S1–S2).

## Results

Earthquake hypocenters and seismic attenuation anomalies (Fig. 3a, left hand column) are compared with surface geomorphology^30, 31^, gravimetry^32^ and Vp/Vs ratios^24^ (Fig. 3a, right hand column). Microearthquakes delineate existing NE-SW and NW-SE fractures and faults^30^, in particular (1) the NW-SE trending La Starza marine terrace (Fig. 3a, upper right-hand panel) and (2) a NW-SE trending fault offshore Mt. Nuovo (Fig. 3a, left-hand column). Two high-attenuation anomalies, similar to those retrieved by De Siena *et al*.^28^, connect Pozzuoli to the Solfatara and Mt. Nuovo craters at 1.2 km depth, following La Starza marine terrace and the associated NW-SE and WNW-ESE extending fractures (Fig. 3a, first row). During the unrest, the Pozzuoli-Solfatara anomaly was seismically active down to the average depth of 2.2 km. Microearthquakes were nucleated under Pozzuoli and below 2.2 km depths and reached vents active in the past 5 kA (white arrows), south of the Solfatara crater (e.g., Fig. S6). The Pozzuoli-Mt. Nuovo high-attenuation anomaly was seismically active only along La Starza.

The microearthquakes imposed on the horizontal attenuation tomograms at depths of 2.5 km, 3.2 km, and 4.25 km (Fig. 3a, left-hand column) are all located below the maximum inferred depth of the caprock^14^ and drawn as orange circles. The 2.5 km deep attenuation model is compared to gravity anomalies (Fig. 3a, second row^32^). Circular white arrows are imposed on the tomograms where the seismicity crosses Mt. Nuovo and connects Pozzuoli to the trace of a NW-SE trending fault offshore (2.5 and 3.2 km depths). This active fault crosses the low-gravity and high/average attenuation portion of the caldera. Seismic attenuation is very low onshore, except under Solfatara. Offshore Pozzuoli and Solfatara, high attenuation anomalies follow the aseismic trace of a second NW-SE trending fault and generally correspond to the high-gravity eastern portion of the caldera.

The 3.2 km deep horizontal tomograms in Fig. 3a (third row) show a single seismically active high-attenuation domain, spatially related to $\frac{{V}_{p}}{{V}_{s}}$ ratios as low as 1.2. The small arrows pointing to ENE and WSW show the direction of microearthquake propagation observed on April 1, 1984. The lowest $\frac{{V}_{p}}{{V}_{s}}$ and high attenuation values are located NW of the best fit centre of the 1982–1984 inflation based on the simultaneous inversion of surface deformation and gravity data^8^ (red circle). High $\frac{{V}_{p}}{{V}_{s}}$ ratios (>2) offshore Mt. Nuovo clearly delineate a seismic NW-SE trending fault and correspond to a zone of scarce resolution in the attenuation model, west of a low-attenuation anomaly.

At 4.25 km, the only high-attenuation anomaly is aseismic, corresponds to $\frac{{V}_{p}}{{V}_{s}}$ between 1.6 and 2, and shows a preferential axis perpendicular to the WSW-ENE extension of the deep seismicity (Fig. 3a, left, 4.25 km). Although ray density has improved inside these deep volumes with respect to previous studies^28^ (Fig. S2a–c), the high-attenuation anomaly is unreliable south of 4518000 UTM/WGS84, while its NW-SE trend could be induced by the scarce azimuthal illumination on the structures (Figs S3–S5). The 3D isosurfaces in Fig. 3b delineate the main attenuation anomalies below 2.2 km depth and comprise: (1) a 4–4.5 km deep aseismic high-attenuation anomaly offshore Pozzuoli (Fig. 3b, orange isosurface), located just below the main deformation and gravimetric anomalies, related to magma accumulation and modelled for this unrest (yellow ellipsoid^11^ and red sphere^8^); (2) a 3–4 km deep high-attenuation and low-$\frac{{V}_{p}}{{V}_{s}}$ seismically active volume under Pozzuoli (red isosurface), connected to the deeper high-attenuation anomaly; (3) a low-attenuation 2.2–2.8 km deep layer (cyan isosurface) separating this last domain from the maximum inferred depth of the caprock^14^.

Microseismic patterns active in 1983 (Fig. 4a–c) and 1984 (Fig. 5a–d) are imposed on geomorphological maps and plotted on vertical attenuation tomograms in different time periods (horizontal scale showing days and months), using both the maximum likelihood hypocentres (black and orange circles) and the non-linear location PDFs (red dots)^33^. The area intersected by the Mt. Nuovo eruption^1, 4^ (yellow circumference on all panels, Figs 4 and 5) shows a consistent seismicity only (1) below the maximum depth of the caprock (Fig. 5c,d) and (2) after March 1984 (Fig. 5a). The deformation anomalies (red circle and yellow ellipse) are plotted on each panel. In the vertical tomogram of Fig. 4c (AA’) the dashed horizontal black lines delineate the layer between the maximum depth of the caprock^14^ and the top of the 3–4 km deep high-attenuation anomaly. In Fig. 5b,c, the white ellipsoidal contours show the volumes ruptured on April 1, 1984. The *CO*_2_/*H*_2_ *O* and *CH*_4_/*H*_2_ *O* ratios collected at the Solfatara crater over time (Fig. 2) give a geochemical perspective on the time-dependent dynamics of the unrest and are compared to the spatial seismic patterns. In particular, we test the seismic patterns against models of repeated closed-system decompressions, physically simulated by geochemical studies^16, 17^ below 2 km (the minimum depth of each swarm) as the cause of *CO*_2_-bearing fluid migrations along the Pozzuoli-Solfatara axis.

### The high attenuation anomalies between depths of 3 km and 4.5 km

The seismic characteristics of the high-attenuation domain between depths of 3 and 4 km (Fig. 3a, 3.2 km) are compatible with the presence of fractured over-pressured gas-bearing formations and wave-induced flow under Pozzuoli^9, 14, 25, 34^. At the corresponding pressures and temperatures^35^ water- or brine-bearing supercritical *CO*_2_- and *CH*_4_ would be present^34, 36^. Supercritical fluids are characterised by high attenuation^37^, low velocity ratios^38, 39^ (see Fig. 3a, 3.2 km, right) and high microseismic activity^9^. Their presence is supported by rock composition, temperature and pressure conditions data in this depth range^35^. Above 3.2 km depth, microearthquakes propagate out of the reservoir and reach the vents south of Solfatara following La Starza marine terrace (Fig. 3a, first row, white arrows). This is a direct seismic evidence of the continuous *CO*_2_- and *CH*_4_-bearing fluid migrations acting between the reservoir and the Solfatara crater^14, 17, 40^ during the unrest.

We look at the spatial and temporal relations of the 4–4.5 km deep high-attenuation anomaly with geomorphological and geophysical results (Figs 3a, 4a–c and 5a–d, S1, and S6) to understand if (1) its existence is justified and (2) the dynamics of the unrest (seismic ruptures and deformation patterns) may clarify its nature. The main seismic swarms in 1983 (Fig. 4b, September-October) and 1984 (Fig. 5b, April 1) were located just NW and centred 1 km above the high-attenuation anomaly (orange ellipsoid, same panels). The anomaly was aseismic and corresponded to $\frac{{V}_{p}}{{V}_{s}}$ ratios between 1.6 and 2 (Fig. 3a, 4.25 km). Its top was located 0.5–1 km below the main deformation sources proposed at Campi Flegrei for this and recent unrests^2, 8, 11, 41, 42^ (Fig. 4b, yellow ellipsoid and red circle). The trend of the anomaly (NW-to-SE) was similar to the the strike of the main fault offshore Pozzuoli^30^, plotted on all maps, and parallel to the main regional extensional direction. Finally, the anomaly was comprised in the NW-SE boundary between the southwestern low-gravity and northeastern high-gravity caldera portions (Fig. 3a, gravimetry)^32^.

The interaction of the high-attenuation volume with the upper reservoir under Pozzuoli, connected to it (Fig. 3b), can be better understood studying the swarm of 202 vertically aligned micro-earthquakes striking the reservoir on April 1, 1984 (Fig. 5b). This is the date when the continuous rise in deformation during the unrest showed an out-of-trend increase^5, 12, 43^. This is also the date when the 5–7 month long decrease in *CH*_4_/*H*_2_ *O* stopped and an increase in *CO*_2_/*H*_2_ *O* started (Fig. 2). The lateral propagation of the cumulative microearthquakes towards WSW and ENE during this day (supplementary video) was previously inferred by the study of the fault mechanisms of the same swarm^23^. The hypocentral area intersected the reservoir and opened in a direction perpendicular to the NW-SE extension of the high-attenuation anomaly (Fig. 3a, 3.2 and 4.25 km, arrows). The directivity of the rupture, based on a dataset of 26 earthquakes deeper than 2.2 km, is estimated to be NW, with 90% preference over SE (see Fig. S1). Any input from depth producing the April 1, 1984, swarm opened the reservoir and came from SE, where the deformation and gravity anomalies and the 4–4.5 km deep high-attenuation anomaly extended.

### The spatial correlation between deformation and seismic sources

From January to early September 1983, the maximum likelihood hypocentres formed two main clusters north-northwest of Pozzuoli and south of Solfatara, between depths of 2.2 and 4.5 km and above 2.5 km depth, respectively (Fig. 4a, AA, BB’). While the deep Pozzuoli cluster corresponded to the oldest vent in the centre of the caldera, the south-Solfatara seismic cluster comprised on a map the most recent volcanic vents (Fig. 3a, geomorphology, white arrow). The deeper cluster was located NW of the 4–4.5 km deep high-attenuation anomaly and bordered/crossed the 3.5–4 km deep NW-SE oriented deformation ellipsoid^11^ (Fig. 4a, yellow ellipsoid). The seismic zones were seemingly elongated WNW-ESE, the direction of regional extensional stress^2^. The volumes comprising the deep deformation and high-attenuation anomalies were aseismic and embedded in, or just above, the caldera basement^14^. The results thus point to the existence of a unique high-attenuation source of deformation and seismicity during the 1983–84 unrest. This structure had a principal horizontal axis NW-SE or WNW-ESE, parallel to the present caldera extensional direction^2, 43^ and the direction of the magma transfer preceding the last eruption^4^. The domain intersected at the surface the only volcanic vent active 9.5–15 kA ago in the centre of the caldera (Fig. 3a, green dot near Pozzuoli) and past volcanic activity may have played a role in the burial of such a structure^12, 16^.

Amoruso *et al*.^11^ show that the main deformation source was paired with a small, secondary, punctual deformation source under Solfatara (Fig. 4a and Fig. S6, yellow circle). Our results confirm that this secondary source was located in an aseismic volume at the boundary of the seismic cluster south of the Solfatara crater, in a region of high attenuation corresponding to the upper hydrothermal system^44^. In 1983, microearthquakes spread from the Pozzuoli swarms, upwards to the southern border of the Solfatara deformation source, inside the caprock (Fig. S6). The seismic patterns and attenuation anomalies thus support the existence of paired seismic and deformation sources characterised by high attenuation. Extensive interdisciplinary literature supports the claim that NW-SE and WNW-ESE oriented tectonic structures may also drive more recent unrests at Campi Flegrei. These include earthquakes occurring along a ~NW-SE preferred strike^45, 46^ and normal NW-SE faults moving in response to a NNE-SSW to NE-SW extension^47^. The two paired deformation sources^11^ were active after 1984, until 2010. Finally, an ellipsoidal-shaped magmatic body with principal axis NNW-SSE and centre offshore Pozzuoli was detected at 4–5 km depth by using data from strainmeters and tiltmeters recorded during the 2011–2013 deformation unrest^42^.

### The spatial and temporal correlations between geochemical data and modelling and seismic results

From September-October 1983, no relevant geochemical variations (Fig. 2) were paired with repeated vertically aligned swarms just NW of the Pozzuoli City centre. The seismicity beneath Pozzuoli culminated on September 10 and 19 as well as October 2 and 13 (Fig. 4b). The swarms intersect (1) the 3–4 km deep, high-attenuation zone under Pozzuoli and (2) the 2.2–2.8 km deep, low-attenuation layer (Fig. 3b). After each swarm, seismicity propagated east towards Solfatara (Fig. S6). By the end of 1983, the Pozzuoli and Solfatara clusters acted in the same volumes defined by the January-September seismicity (compare Fig. 4, panels (a) and (c)).

Strong increases of *H*_2_ *S*/*CO*_2_ were observed by Moretti *et al*.^16^ between June and December 1983 and modelled by the same authors using two magmatic sources at 4 km and 8 km depths. In September-October 1983, repeated closed-system decompressions were physically simulated by Chiodini *et al*.^17^ below 2 km as the cause of *CO*_2_-bearing fluid migrations along the Pozzuoli-Solfatara deformation axis (dashed vertical lines in Fig. 2). In October 1983, the authors modeled the source of the geochemical variations driving volcanic unrest to critical state as injections produced by a deeper hot source. Such fluid migrations caused geochemical, heat, and fluid-flux variations in the shallower hydrothermal systems and, from a geochemical perspective, rule the unrest behaviour^16, 44, 48^. The seismic swarms observed in September-October 1983 (Fig. 4b) thus locate flux variations and injections inside the supercritical fluid reservoir and below the caprock. Vanorio and Kanitpanyacharoen (2015)^14^ suggest that a second *CO*_2_-bearing fluid phase under supercritical conditions is originated at 3 km depth causing the 1983–84 uplift. All models and experiments, from wave-induced fluid flow to wave induced gas exsolution dissolution^37, 38, 49^, confirm that the presence of gas (or in general a supercritical fluid phase) also lowers seismic wave attenuation of shear waves. The attenuation model presents the above-mentioned low-attenuation layer between depths of 2.2 and 2.8 km, just above the 3–4 km deep reservoir and below the maximum depth of the caprock (Fig. 4c, between dashed lines).

### Opening of the Pozzuoli reservoir and halting of the seismic unrest

Microearthquakes connected the 3–4 km deep reservoir with the fumaroles at Mofete/Mt. Nuovo (the location of the last eruption, Fig. 1) from the start of 1984 (Fig. 5a, compare different time periods). The Pozzuoli-Mt. Nuovo connection was seismically active below 2 km depth and followed the caldera-bounding and NW-SE striking offshore normal faults west of Pozzuoli^21, 50^. These faults were thus aseismic throughout January 1984 and activated between February 10 and March 31, 1984 (Fig. 5a). Lateral earthquake migration remained inefficient until the main seismic swarm on April 1, 1984 (Fig. 5b shows shallow and deep microearthquakes on different panels). During the day (supplementary video), the 202 micro-earthquakes comprised in the swarm stroke first the centre of the reservoir, then progressively spread towards WSW (offshore Mt. Nuovo) and ENE (Solfatara - Fig. 5b, arrows). The focal mechanisms of the deep micro-earthquakes show the migration of deeper materials towards the surface in an ellipsoidal domain (Fig. 5b,c) having principal axis oriented NW-SE^23^, the same directions of the principal axis of the deformation ellipsoid, the high-attenuation anomaly, and the main fault offshore Pozzuoli. The directivity study (Fig. S1) shows that the direction of this structurally controlled input was NW.

This input stroke the 3–4 km deep reservoir, releasing *CO*_2_-bearing fluids (Fig. 2, April 1) that rapidly reached Solfatara volcano from SE. Between April 2 and May 27 (red dots and white ellipsoid, Fig. 5c) the ellipsoidal rupture region became aseismic (supplementary video) while deformation persisted even if dampened^43^. The depth range (~2.75 km) is consistent with that reconstructed by Woo and Kilburn^2^ for the intrusion of a small magmatic dyke. The period was followed by a relevant change in the seismic patterns. Between May 28 and December 31 (Fig. 5d) microearthquakes spread across the caldera crossing Mt. Nuovo below the caprock and following pre-existent tectonic structures (Fig. 5c, orange circles). The stress released by the opening of this pathway halted the seismic unrest.

## Discussion and Conclusions

Although the resolution of the attenuation model is poor at 4.25 km depth, our tests indicate the existence of a 1 km-wide area of high attenuation SE of Pozzuoli (Figs 3a,b and S3–S5). The NW end of the anomaly lies beneath the inferred inflation centre at 3.2 km depth (Fig. 3b)^8, 11^. Given the geothermal gradient at Campi Flegrei (~200 *K*/*km*), rocks at 4.5 km depth are ductile, although under limited stresses, thus outside the overlying sismogenic layer^51^. The range in $\frac{{V}_{p}}{{V}_{s}}$ ratios observed below 4 km depth just offshore and east of Pozzuoli is 1.6–2 (Fig. 3a). As geochemical studies model the shallowest magmatic source of unrest at around 4 km^16^, the high-attenuation zone represents the most likely volume where *H*_2_ *O* undersaturated and *CO*_2_ saturated magma was stored at Campi Flegrei in 1983–84^2, 8, 42, 44, 52^. If at near-solidus temperature and in the absence of melting^53^, the zone was instead only heated by magmatic sources in contact with it, but outside the study imaging range. In both cases, a feasible cause of the April 2-May 27 aseismic slip under Pozzuoli is an intrusion at ~2.75 km depth^2, 8^ in the 3–4 km deep supercritical fluid reservoir on April 1, 1984.

An alternative explanation for the 3–4.5 km deep high-attenuation system (Fig. 3b) is the onset of wave-induced flow attenuation in reservoirs of multiphase magmatic fluids^37^. This mechanism attenuates P- and S-waves in the presence of heterogeneous saturation^36^ and would better explain the relatively low $\frac{{V}_{p}}{{V}_{s}}$ characterising both anomalies^9^. In this setting, degassing due to changes in pressure and temperature created *CO*_2_ pockets enhancing uplift^14^. Aseismic slip following an injection of fluids along faults under Pozzuoli^54^ on April 1, 1984, could then explain the following extended aseismic period.

Our results support a model where either a caprock^14^ or cooled intrusions^12, 26^ act as a barrier for the fluids released by intrusions/injections from a deeper magmatic source under Pozzuoli. These intrusions/injections enhanced uplift in the centre of the caldera with the additional stress released via active structural pathways leading to Solfatara until the April 1, 1984 swarm. After May 27 and until the end of 1984 (Fig. 5d and supplementary video) stress was released west of Pozzuoli following pre-existent but previously aseismic WNW-ESE and WSW-ENE tectonic structures. Fluids migrated below the caprock towards the Mofete and San Vito geothermal reservoirs (Fig. 1), where they were extracted by AGIP and ENI (two Italian energy retailers)^9, 14^. Fluid migration to the location of the last eruption (yellow circle, Figs 4–5) and further offshore induced fluid saturation of the rocks in the hydrothermal reservoirs, halting the unrest. These results restrict the transition from elastic to plastic behaviour at the volcano^46^ to the period April-December 1984.

The temporal and spatial correlations we observe between seismic (Figs 3–5), tomographic (Fig. 3a,b), geochemical (Fig. 2)^16, 17^, and deformation^2, 8, 11, 14^ models show that the high-attenuation and deformation area offshore Pozzuoli was the most feasible hot feeder for the seismic, deformation, and geochemical 1983–84 unrest. Future research must focus on better characterising the features and nature of these volumes, at a time when geophysical and geochemical signals indicate that the volcano is reactivating^17, 55^. The structures and fluid-induced dynamics we describe are a template for future unrests at this and other calderas, where they may improve assessment of volcanic hazard.

## Methods

### Micro-earthquake locations, directivity analysis

Our original dataset comprises more than 200.000 pickings of P- and S-waves corresponding to 10410 events recorded in 1983–1984 by 15 three-component seismic stations deployed by the University of Wisconsin and 20 stations part of the permanent network of the Osservatorio Vesuviano and Aquater AGIP^20, 24^. A final dataset of 81.636 high-quality pickings was selected in order to relocate 2406 microseismic events using the NonLinLoc software^33^ and the 3D P- and S-wave velocity models of Battaglia *et al*.^24^. These velocity models were obtained using active shots data to stabilise the inversion in the shallowest Earth layers (down to two km). Below 2 km, the velocity models show no relevant difference with respect to those of Vanorio *et al*.^9^, which use only passive seismicity recorded during the 1983–84 unrest. In Fig. 3a, $\frac{{V}_{p}}{{V}_{s}}$ panels at 3.2 and 4.25 km, we shade areas of low or no resolution as reported by these two studies.

The maximum likelihood solutions of the complete, non-linear location PDF (Figs 4–5 red dots, 1000 samples for each event) were obtained using the Oct-Tree grid search algorithm in NonLinLoc^33^. The 2406 maximum likelihood solutions and PDF in the final dataset have the following specifics: a minimum of either eight P-phases or six P-phases and two S-phases available; a root-mean-square error lower than 0.30; an azimuthal gap lower than 180 °*C*; a single maximum in the PDF; the main axis of the 68% Gaussian ellipsoid smaller than 4 km.

On April 1, 1984, 202 microearthquakes are recorded in a single cluster at the northwestern end of a fault of known strike^30^ (NW-SE, Fig. 3a, geomorphology, the cluster is reported in Fig. 5b). During the day (supplementary video), locations spread from the fault towards west (offshore Mt. Nuovo) and east (north of Solfatara - Fig. 3a, white arrows). The final rupture is approximately perpendicular (WSW-ENE) to the strike of the offshore NW-SE fault. This is confirmed by the source mechanisms of the micro-earthquakes deeper than 2.2 km, which show opening of this fault in an ellipsoidal domain with principal axis oriented NW-SE^23^.

To understand the preferential direction of the rupture (either NW or SE) we apply the directivity analysis developed by Kane *et al*.^56^ to the April 1 1984 cluster. This will constrain the location of the structure producing the rupture (SE or NW of the cluster, respectively). The directivity is computed by looking for azimuthal differences in spectral amplitudes of S-wave displacement spectra (Fig. S1) and quantified as the log-difference of the mean spectral amplitudes to the northwest versus the southeast directions over the 15–24 Hz frequency band^56^.

### Seismic attenuation imaging, geomorphology and result display

The seismic dataset recorded between January 7,1984 and April 14, 1984 provides a S-wave 3D attenuation image of the structures producing the strongest seismic unrest (Fig. 3a, left-hand column). The coda-normalised amplitudes of 3-component waveforms corresponding to the same 853 source-stations pairs used in De Siena *et al*.^28^ are inverted for the inverse S-wave quality factor (${Q}_{s}^{-1}$) using the open-access code MuRAT^29^ and the new source-station configuration derived by the use of NonLinLoc^33^, both readily available online. MuRAT inverts the coda-normalised body-wave intensities filtered at 6 Hz and averaged over different components for ${Q}_{s}^{-1}$ in a grid of 0.5 km spaced nodes. The locations and directivity analysis improve the attenuation model in the sense that (1) spatial uncertainties are better assessed with respect to prior analyses, particularly the reliability of sources offshore, thanks to the use of PDFs; (2) they now better illuminate depths between 3.5 km and 4.5 km, and (3) before the inversion, we can now correct all waveforms produced by the April 1, 1984 swarm for source directivity.

To prove the change in depth illumination with respect to De Siena *et al*.^28^ we compare the ray hit-count for blocks between depths of 4 and 4.5 km for the two studies (Fig. S2a). The increase is especially relevant for the aseismic zone offshore Pozzuoli (from 10 to 35 rays), where the deepest high-attenuation anomaly is located (orange contour). The correction due to the estimate of directivity (Fig. S1) is: (1) a multiplier of 3 for energies recorded at stations NW of the swarm and in the area (energies three times larger than SE); (2) a factor of 2 for energies recorded NE and SW of the swarm and in the area (energies two times larger than SE).

After correcting the coda-normalised energies for these factors, the ill-defined underdetermined problem (853 data for 1279 model parameters) is first corrected by assuming that each of the model parameters with scarce sampling (less than 4 rays) is equal to the average ${Q}_{s}^{-1}$ (0.0205 ± 0.0029, Fig. S2b). This parameter is obtained by a preliminary least square inversion of all coda-normalised energies again travel time. This leads to a 853 data by 546 model parameter problem, which is assessed using resolution and spike tests (Figs S3–S5). The reduction in damping parameter with respect to the previous Tikhonov inversion is relevant (from 0.1127^28^ to 0.0346, Fig. S2c). The sum of the root mean square of the model residuals is now 7.23 against a previous estimate of 12.50^28^.

We assess both reliability and resolution of the model obtained in the final inversion following all procedures described by Rawlinson and Spakman 2016^57^. Ray-path geometry is inherited from the model constrained by the observations, i.e., we use the same forward matrix for obtaining the results and test outputs. In Figs S3–S5 we show the results of the checkerboard test, where input anomalies are blocks having side 2 km, and of two different synthetic anomaly tests, respectively. The 2 synthetic anomaly tests (Fig. S4) are performed to check the effective recovery of 2 km-side high-attenuation blocks (1) mimicking the 3.2 km deep (2) the 4.25 deep high-attenuation anomalies shown in Fig. 3a, left, and of a layer of low attenuation (Fig. S5) sandwiched between high-attenuation structures. Recovery of all anomalies is mostly unchanged with respect to De Siena *et al*.^28^ above 3 km. At 3.2 km depth and especially offshore (compare Fig. S4 with Fig. 3 of De Siena *et al*.^28^) the recovery of the high attenuation anomaly is improved due to the new source-ray configuration, with small artefacts created above and, especially, below it.

The checkerboard test (Fig. S3) shows poor resolution at a depth of 4.25 km, with all high-attenuation anomalies strongly smoothed. This depth range was neither discussed nor tested in De Siena *et al*.^28^ as it showed no relevant variations and tests did not reproduce any of the input anomalies. The synthetic anomaly tests (Figs S4–S5), however, shows that, while artefacts are likely at depths below 4 km and outside the central area of the model, the central part still reproduces the high attenuation features. In addition, no main directionality (e.g., NW-SE) is visible in the output, with the block generally keeping its symmetrical features (Fig. S4).

In Fig. 3a, faults and coastline are transformed into a shape file and imported in Voxler 3.0^©^. Attenuation maps, $\frac{{V}_{p}}{{V}_{s}}$ maps, microseismic hypocenters, and isosurfaces (Fig. 3b) were obtained using Voxler 3.0 using the 3D data provided in the submission, the velocity model of Battaglia *et al*.^24^ and a distant weighting interpolation method of second order for tomograms. Deformation/gravity anomalies reproduce the general features of the anomalies described by the corresponding studies^8, 11, 32^. The layout of the figure, the axes for each panels, the deformation/gravity anomalies, and the arrows have been created using Photoshop CS^©^. They are then imposed on all maps reproduced in this and following figures. In Fig. 3a,b we mask areas of limited or no resolution in the model. For the anomaly at 4.25 km depth we only unmask the area where the synthetic test shows adequate recovery, i.e., the central domain. The domain is affected by higher uncertainties than, e.g., the high-attenuation anomaly at 3.2 km depth. In order to understand its effective reliability we compare its spatial relationship with seismic patterns and different geophysical (deformation), geomorphological, and geochemical parameters in the main text.

### Data availability

The datasets analysed during the current study are available in the PANGAEA repository, doi:10.1594/*PANGAEA*.875535.

## Electronic supplementary material

Supplementary Video

Supplementary Figures
