# Supplementary material for: Source and dynamics of a volcanic caldera unrest: Campi Flegrei, 1983–84
Source: Sci Rep. 2017 Aug 14;7:8099. doi: 10.1038/s41598-017-08192-7 (PMC5556014; doi:10.1038/s41598-017-08192-7)
Supplement: Supplementary file 2 — Supplementary Figures [file 41598_2017_8192_MOESM2_ESM.pdf]

Source and dynamics of a volcanic caldera unrest: Campi Flegrei, 1983-84

Luca De Siena[1]*, Giovanni Chiodini[2], Giuseppe Vilardo[3], Edoardo Del Pezzo[3,4], Mario Castellano[3] Simona Colombelli[5], Nicola Tisato[6], Guido Ventura[7,8]

[1] University of Aberdeen, School of Geosciences, Dept. Geology and Petroleum Geology, Meston Building, King's College, Aberdeen AB24 3UE Scotland, UK
[2] Istituto Nazionale di Geofisica e Vulcanologia, Sezione di Bologna, Via D. Creti 12, 40128 Bologna, Italy
[3] Istituto Nazionale di Geofisica e Vulcanologia, Sezione di Napoli-Osservatorio Vesuviano, Via Diocleziano 328, 80124 Napoli, Italy
[4] Instituto Andaluz de Geofisica, Universidad de Granada, Calle Prof. Clavera, Campus Universitario de Cartuja, Granada, Spain
[5] Dept. of Physics, University of Naples Federico II, Napoli, Italy
[6] The University of Texas at Austin, Jackson School of Geosciences, Department of Geological Sciences, 2275 Speedway Stop C9000, Austin, TX 78712
[7] Istituto Nazionale di Geofisica e Vulcanologia, Sezione di Roma, Via di Vigna Murata 605, 00181 Roma, Italy
[8] Istituto per l'Ambiente Marino Costiero, CNR, Napoli, Italy

Supplementary Materials

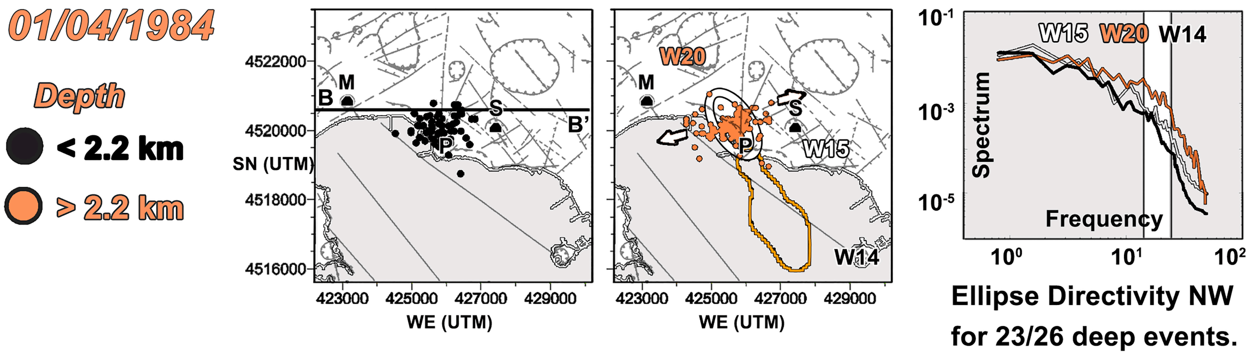

Supplemental Figure 1. April 1 1984 seismic cluster and NW directivity. Shallow (black circles) and deep (orange circles) microearthquakes recorded on April 1. Three seismic stations are used to study the directivity of the deep April 1 cluster: W20 (orange), W15 (white) and W14 (black). The strike of the fault is NW-SE, hence we assume the rupture is produced at the NW end of the fault just offshore Pozzuoli, as also deduced by the study of the focal mechanisms of the corresponding earthquakes (De Natale et al 1995). Arrows show the aperture of the rupture below the caprock (compare with video SV1) during the day. The stacked average seismic spectra are shown on the right. The frequency band chosen for the analysis is shown in white. The average spectra have been obtained by using Matlab R2014b © (https://uk.mathworks.com/campaigns/products/ppc/google/matlab-trial-request.html?s_eid=ppc_29742998122&q=matlab). Maps created with Voxler 3.0 (http://www.goldensoftware.com/products/voxler). Layout and axes created with Photoshop CS6 © ((http://www.adobe.com/products/photoshop.html).

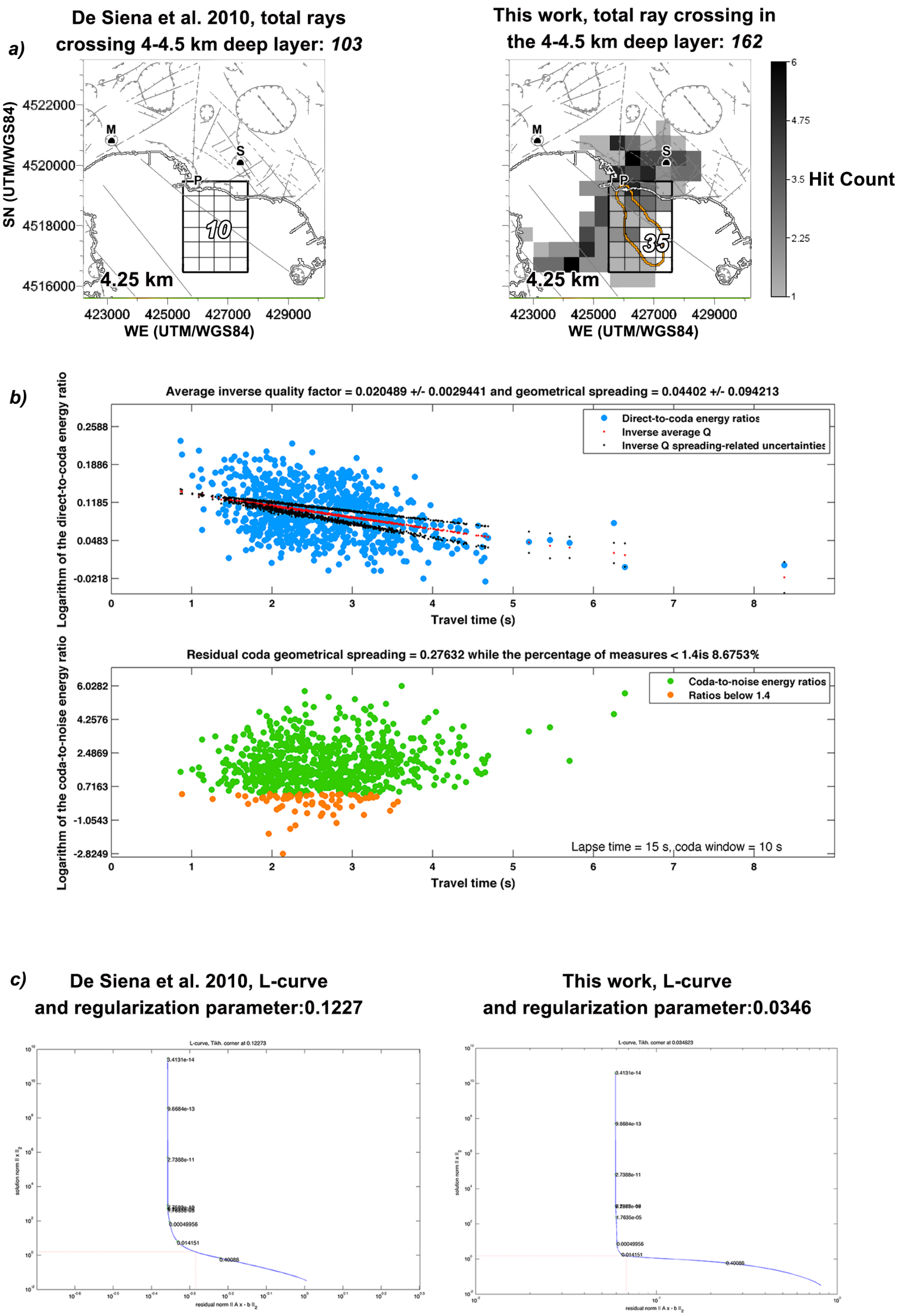
a)
De Siena et al. 2010, total rays crossing 4-4.5 km deep layer: 103
This work, total ray crossing in the 4-4.5 km deep layer: 162
SN (UTM/WGS84)
4522000
4520000
4518000
4516000
M
S
P
10
35
4.25 km
423000 425000 427000 429000
WE (UTM/WGS84)
Hit Count
6
4.75
3.5
2.25
1
b)
Average inverse quality factor = 0.020489 +/- 0.0029441 and geometrical spreading = 0.04402 +/- 0.094213
Direct-to-coda energy ratios
Inverse average Q
Inverse Q spreading-related uncertainties
Logarithm of the direct-to-coda energy ratio
0.2588
0.1886
0.1185
0.0483
-0.0218
Travel time (s)
Residual coda geometrical spreading = 0.27632 while the percentage of measures < 1.4is 8.6753%
Coda-to-noise energy ratios
Ratios below 1.4
Logarithm of the coda-to-noise energy ratio
6.0282
4.2576
2.4869
0.7163
-1.0543
-2.8249
Lapse time = 15 s, coda window = 10 s
Travel time (s)
c)
De Siena et al. 2010, L-curve and regularization parameter:0.1227
This work, L-curve and regularization parameter:0.0346

Supplemental Figure 2. Comparison between previous and new inversions.

a) Comparison between rays crossing the 4-4.5 km deep layer. Inside the grid offshore Pozzuoli we show the number of rays crossing the aseismic area in the two studies. The high attenuation anomaly only appears in the present work, while no relevant anomaly was present in the previous study at these depths. The grid shows the 24 cells comprised in the aseismic area, where the inversions have been performed in the two studies. This grid has been plotted in Matlab R2014b© (https://uk.mathworks.com/campaigns/products/ppc/google/matlab-trial-request.html?s_eid=ppc_29742998122&q=matlab) and imposed on the geomorphological map. The total number of rays shown on the grid is the sum of all the rays crossing the grid for the two studies. ESRI ArcGIS 10.0 (https://www.esri.com/training/catalog/5763042b851d31e02a43ed4d/using-arcmap-in-arcgis-desktop-10/) was used to obtain geomorphological results shown. Both the grid and the numbers have been imposed on the panels using Photoshop CS6 © (http://www.adobe.com/products/photoshop.html). b) The estimate of the average quality factor is obtained by a least square inversion of all data. The first plot shows the fit of the decrease of coda-normalized energies (cyan circles) with increasing travel time (red line). Green and orange circles in the bottom plot show the percentage of coda-normalized energies above a threshold of 1.4, using a coda window taken between 15 s and 25 s from nucleation. c) The L-curve and damping parameter selected by using the MuRAT code for the two inversions. The plots in panels b) and c) are two outputs of the MuRAT code, a free code working in Matlab R2014b©, available at https://github.com/LucaDeSiena/MuRAT.

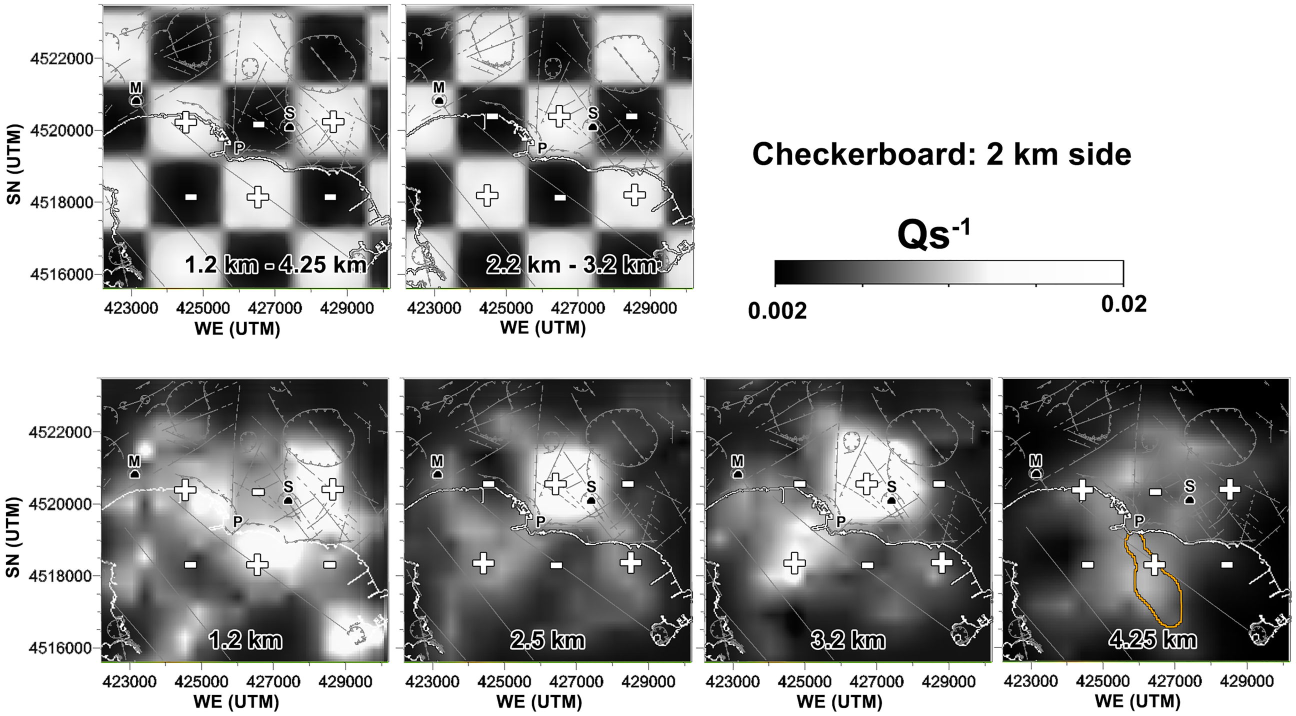

Supplemental Figure 3. Checkerboard test

The checkerboard anomalies are created using the MuRATcode (https://github.com/LucaDeSiena/MuRAT) and plotted using Voxler 3.0 (http://www.goldensoftware.com/products/voxler). Symbols and layout of the figure were created using Photoshop CS6 © (http://www.adobe.com/products/photoshop.html). The interpolation is the same used in Figs. 3-5.

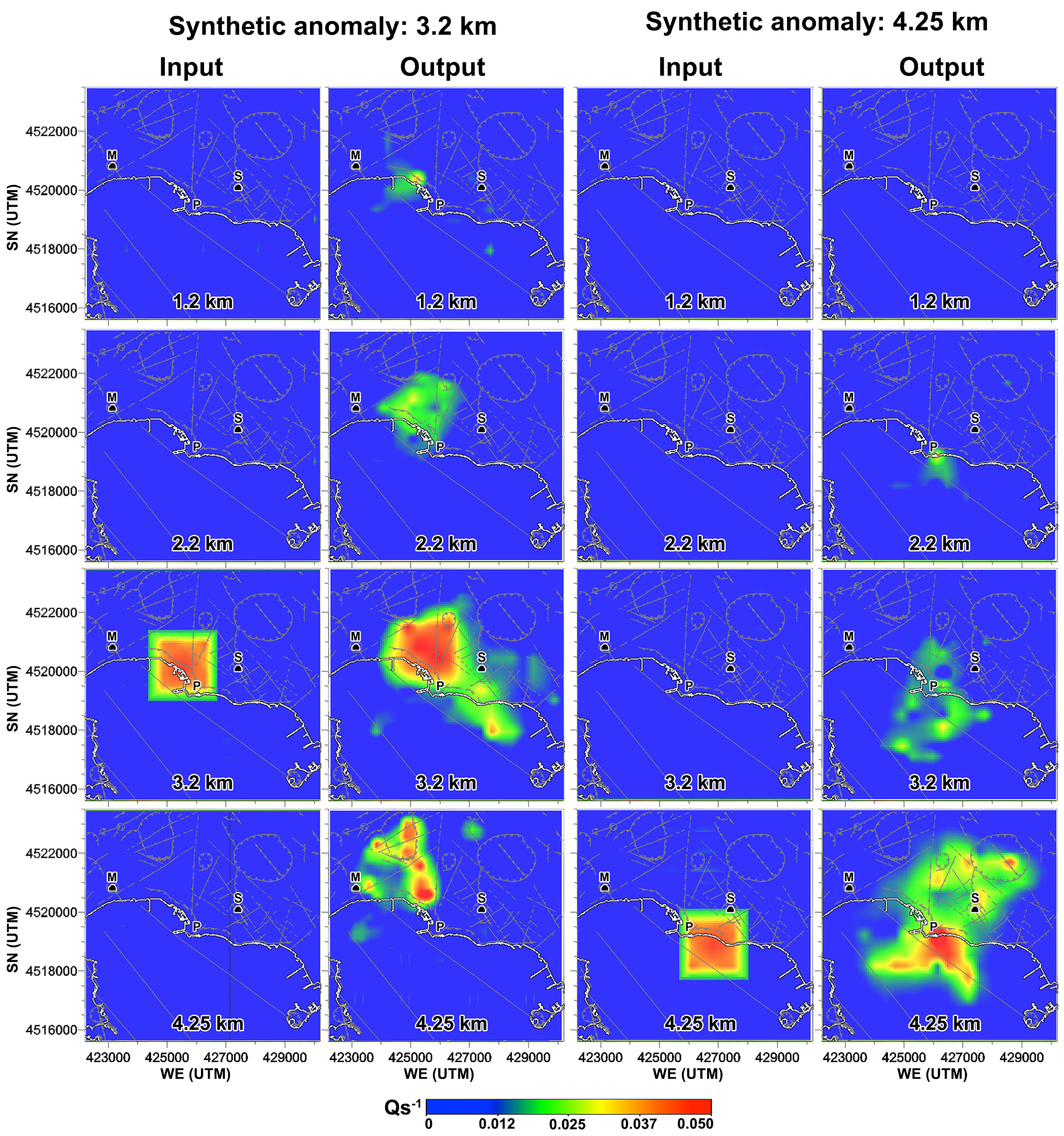

Supplemental Figure 4. Synthetic anomaly tests

The results of 2 spike tests, performed to check the effective recovery of (1) the 3.2 km deep anomaly, interpreted as the a high-attenuation supercritical fluid reservoir, and (2) the 4.25 anomaly interpreted a high-attenuation feeder. The synthetic anomalies are created with the MuRAT code (https://github.com/LucaDeSiena/MuRAT) and plotted using Voxler 3.0 (http://www.goldensoftware.com/products/voxler). Symbols and layout of the figure were created using Photoshop CS6 © (http://www.adobe.com/products/photoshop.html). The interpolation is the same used in Figs. 3-5.

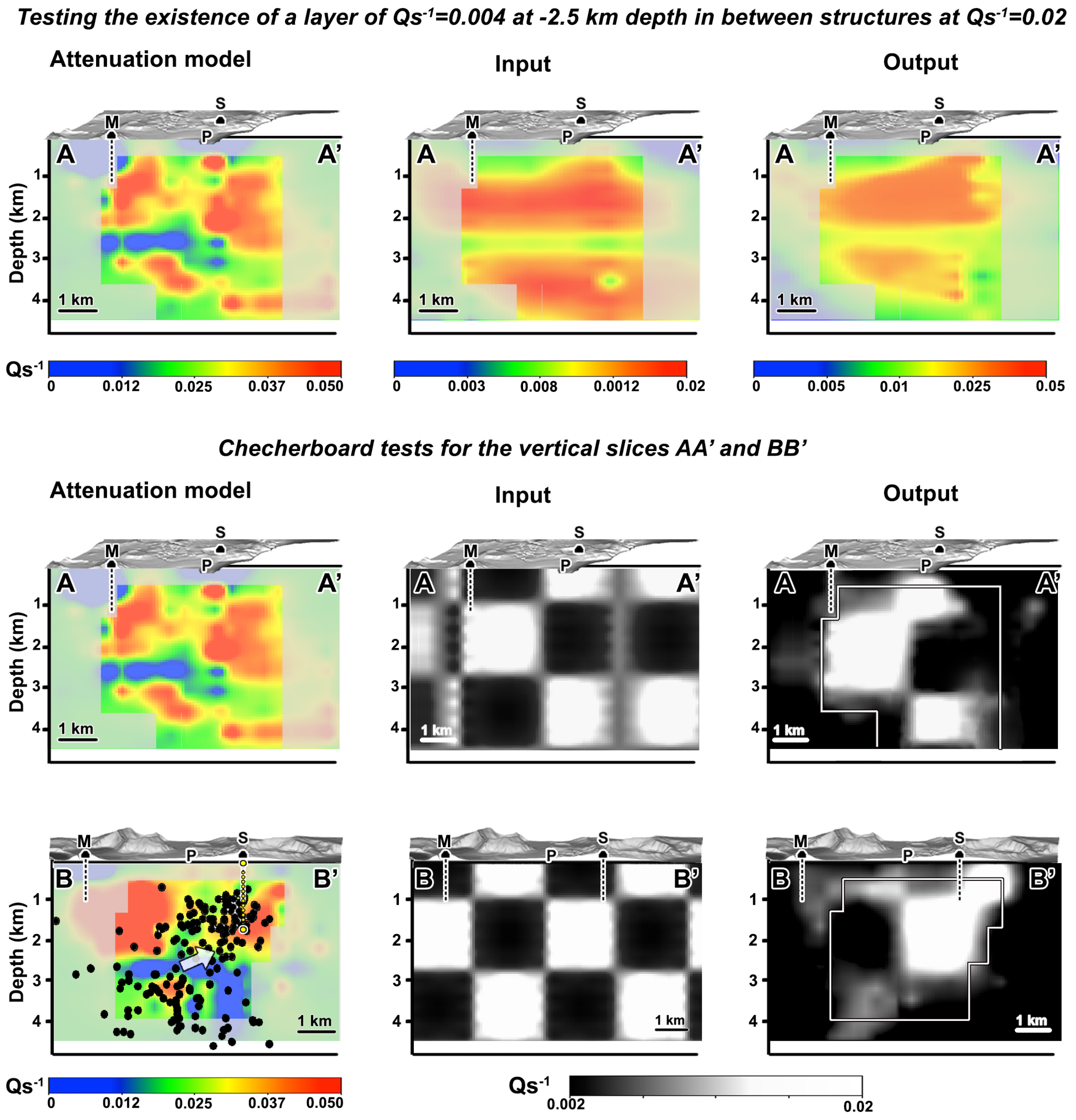

Supplemental Figure 5. Syntetic and checkerboard tests for vertical tomograms. The results of a synthetic anomaly test testing the reliability of the layer of low attenuation sandwiched between 2 high attenuation structures. The results of the checkerboard test are shown on the AA' and BB' transect. The synthetic anomalies are created with the MuRAT code (https://github.com/LucaDeSiena/MuRAT) and plotted using Voxler 3.0 (http://www.goldensoftware.com/products/voxler). Symbols and layout of the

figure were created using Photoshop CS6 © (http://www.adobe.com/products/photoshop.html). The interpolation is the same used in Figs. 3-5.

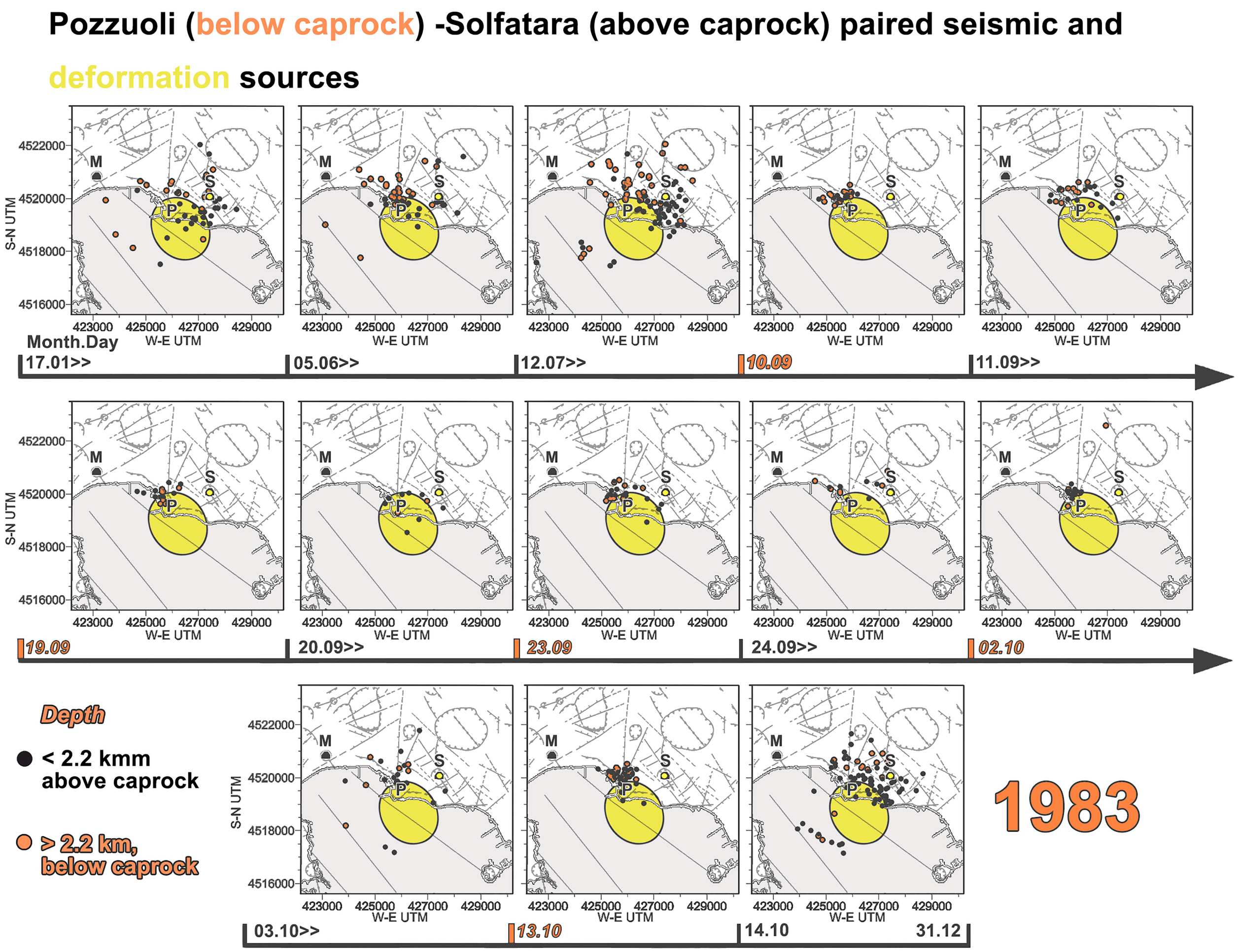

Supplemental Figure 6. 1983 paired deformation and seismic sources. The temporal evolution of the seismicity is shown together with the deformation sources. The two deformation sources (yellow ellipse and circle) are imposed on a geomorphological map on the different panels, which represent the seismic epicentres in different time periods. The two deformation sources are drawn using the data reported in tables 4 and 5 of Amoruso et al. 2014[11]. As symbols and layout, the deformation sources are created in Photoshop CS6 © (http://www.adobe.com/products/photoshop.html). ESRI ArcGIS 10.0 (https://www.esri.com/training/catalog/5763042b851d31e02a43ed4d/using-arcmap-in-arcgis-desktop-10/) was used to plot geomorphological results. Hypocentres were plotted using Voxler 3.0 (http://www.goldensoftware.com/products/voxler).

Supplementary Video. Time-dependent seismicity – April 1- December 31, 1984. Microearthquake locations in three different time periods are over-imposed on the AA' seismic attenuation transect. The background geomorphological map (left) and attenuation transect (right) are the same shown in Fig. 5b and created using the software mentioned in the corresponding caption. Hypocentres are created one by one as single PNG files and used as frames to create a MP4 video with a Matlab R2014b ((https://uk.mathworks.com/campaigns/products/ppc/google/matlab-trial51 request.html?s_eid=ppc_29742998122&q=matlab) self-written script. The video was imposed on the geomorphological map and attenuation transects using Premier Pro 2015.3 (https://blogs.adobe.com/creativecloud/adobe-premiere-pro-and-media-encoder-2015-3-10-4-updates/).
